# A Methodological Framework to Estimate the Site Fidelity of Tagged Animals Using Passive Acoustic Telemetry

**DOI:** 10.1371/journal.pone.0134002

**Published:** 2015-08-11

**Authors:** Manuela Capello, Marianne Robert, Marc Soria, Gael Potin, David Itano, Kim Holland, Jean-Louis Deneubourg, Laurent Dagorn

**Affiliations:** 1 IRD, UMR MARBEC (IRD, Ifremer, Univ. Montpellier, CNRS), Sète, France; 2 Unit of Social Ecology, Universitè libre de Bruxelles (ULB), Bruxelles, Belgium; 3 Fishery technology and biology laboratory, French Institute for the Research and Exploitation of the Sea (IFREMER), Lorient, France; 4 IRD, UMR MARBEC (IRD, Ifremer, Univ. Montpellier, CNRS), Saint Denis, La Réunion, France; 5 Hawaii Institute of Marine Biology, University of Hawaii at Manoa, Kaneohe, United States of America; Pacific Northwest National Laboratory, UNITED STATES

## Abstract

The rapid expansion of the use of passive acoustic telemetry technologies has facilitated unprecedented opportunities for studying the behavior of marine organisms in their natural environment. This technological advance would greatly benefit from the parallel development of dedicated methodologies accounting for the variety of timescales involved in the remote detection of tagged animals related to instrumental, environmental and behavioral events. In this paper we propose a methodological framework for estimating the site fidelity (“residence times”) of acoustic tagged animals at different timescales, based on the survival analysis of continuous residence times recorded at multiple receivers. Our approach is validated through modeling and applied on two distinct datasets obtained from a small coastal pelagic species (bigeye scad, *Selar crumenophthalmus*) and a large, offshore pelagic species (yellowfin tuna, *Thunnus albacares*), which show very distinct spatial scales of behavior. The methodological framework proposed herein allows estimating the most appropriate temporal scale for processing passive acoustic telemetry data depending on the scientific question of interest. Our method provides residence times free of the bias inherent to environmental and instrumental noise that can be used to study the small scale behavior of acoustic tagged animals. At larger timescales, it can effectively identify residence times that encompass the diel behavioral excursions of fish out of the acoustic detection range. This study provides a systematic framework for the analysis of passive acoustic telemetry data that can be employed for the comparative study of different species and study sites. The same methodology can be used each time discrete records of animal detections of any nature are employed for estimating the site fidelity of an animal at different timescales.

## Introduction

Technological advances in biotelemetry provide powerful tools to observe free-ranging animals in their natural environment [1]. Thanks to these techniques, scientists can now study the physiology, behavior and ecology of wild animals in remote areas over long time periods [2]. Just as technological improvements in satellite tracking promoted the development of quantitative movement analysis for many terrestrial species, so did the study of marine species benefit from the parallel growth of acoustic-telemetry technologies and methods to quantify those results [3–7]. Among the available acoustic telemetry techniques, passive acoustic tracking is based on acoustic receivers recording the presence of “tagged” individuals, i.e. individuals equipped with acoustic transmitters. This technique has become a widespread research tool offering a unique opportunity to address both scientific and management questions for many marine species [6, 8–10]. The amount of passive acoustic tracking data is growing rapidly but at the same time little research has been dedicated to the development of methods to analyze such data [11]. Among the diversity of scientific questions addressed through the use of this observational tool, inferring site fidelity in terms of the amount of time spent by the tagged individuals in the vicinity of the acoustic receiver, the so-called “residence times”, is a recurrent objective [4, 10, 12]. Our study aims to provide a methodological framework to support the current subjective methods used to estimate residence times from the presence/absence data produced by an array of acoustic receivers. This is an essential step towards a quantitative comparison of results obtained for different species and study sites.

Estimating residence and absence times demands constructing continuous observations from discrete (both in time and space) acoustic detections (see S1 Fig in the Supporting Information). The discrete nature of the acoustic data derives from the transmission rate and range of the tags, the finite number of receivers and the possibility of missing the acoustic transmissions due to sonic collisions and ambient noise. The first two factors depend on the tag specifications and the way the study site is spatially instrumented. Also, even if a tagged animal is present within the theoretical range of detection of an acoustic receiver and the tag is transmitting, acoustic detections can be missed due to sonic collisions between two or more tags transmitting simultaneously [6]. This can result in misleading interpretations on the presence/absence of the animal. The rate of these collisions depends on the number of tagged fish present around the same acoustic receiver and the specifications of the tags. In addition, ambient noise in the aquatic environment can vary with the time of day and with the environmental conditions, thus affecting the detection characteristics of the acoustic receiver. Current, turbidity, salinity, temperature, bathymetry, and substrate, as well as the quantity of biomass present in the vicinity of the receiver, also influence the rate of acoustic detections [12–15]. In summary, several factors (instrumental, environmental, biological) can affect the ability of a given receiver to accurately detect a tagged animal. The challenge is therefore to determine when a tagged fish is truly absent, i.e., identifying an appropriate temporal scale for the analysis of residence and absence times. Establishing the lower limit of the monitoring period required to detect the presence of a tagged fish constitutes a first application of the methodological approach presented in this paper. As a second application, we consider the issue of analyzing residence and absence times from an ethological and ecological perspective. Establishing a temporal scale for the analysis of acoustic data according to the scientific question of interest is a common practice in fish behavioral studies and marine ecology [16–21]. Fish can make regular diel excursions out of the range of detection (due to feeding, resting or mating behavior, predation avoidance strategies, etc.), causing regular absences of the signal [22–25]. If the main focus of the study is to determine the presence of a fish, independently from these short-term regular patterns of absences, a temporal scale that encompasses these excursions must be chosen to process the data. This temporal scale has thus far been chosen according to the author’s expertise on fish behavior (empirical knowledge). Although the measured behavioral events (e.g. residence times [16, 17, 19, 26], number of visits [18] and synchronicity of departures [23, 27]) are sensitive to the choice of this timescale, the issue of assessing the validity and the sensitivity of the resulting behavioral metrics is tackled in only a few papers (e.g, [27]). In this work, we develop, validate and apply a general method for assessing these temporal scales, taking the case study of two pelagic fish species, bigeye scad (*Selar crumenophthalmus*) and yellowfin tuna (*Thunnus albacares*), tracked in two different arrays of acoustic receivers.

## Materials and Methods

### Methodological framework to estimate site fidelity

#### Definition of continuous residence times (CRT)

The methodological developments presented hereafter generalize an approach originally introduced within the literature on the behavioral ecology of pelagic fish around floating objects (also referred to as Fish Aggregating Devices or FADs), which largely exploit passive acoustic telemetry data [17–19, 23, 27–32]. Ohta and Kakuma (2005) defined a continuous residence time (CRT) as the duration within which a tagged fish was continuously monitored at a specific location without day-scale (> 24 h) absences. This timescale of 24 h was later generalized and referred to as Maximum Blanking Period (MBP, see [27]), which corresponds to the maximum amount of time that is allowed between two subsequent acoustic detections for considering that a fish is still present (or resident) at a particular listening station. Based on this approach, CRTs are defined as time units where the temporal separation between subsequent acoustic detections is smaller than the MBP. In the case of an array of acoustic receivers, the recording of a residence time of a given tagged fish at a receiver *R*_*A*_ starts at the time of the first detection at this receiver (denoted as *t*_0_). When the fish is detected at another receiver (called *R*_*B*_) at time *t*_2_, after being detected for the last time at *R*_*A*_ at time *t*_1_, the CRT at *R*_*A*_ is estimated as *t*_1_ − *t*_0_ and a new residence time at *R*_*B*_ starts at time *t*_2_, regardless of the amount of time elapsing between *t*_2_ and *t*_1_ (*t*_2_ − *t*_1_). However, when the fish is not detected at any other receiver, but is detected again at *R*_*A*_ at *t*_2_, the residence time at *R*_*A*_ is ended at the last detection (*t*_1_) and a new residence time at *R*_*A*_ starts at *t*_2_, each time the temporal interval *t*_2_ − *t*_1_ is larger than the MBP.

#### Definition of the Maximum Blanking Period as a variable

Unlike the previous literature based upon the subjective definition of the MBP, the MBP is considered here as a discrete variable *MBP*_*n*_ whose value is optimized according to the question of interest. To this purpose, the variable *MBP*_*n*_ is defined as:

$$MBP_{n}=n\Delta_{MBP}$$ (1)

where *n* is a positive integer and Δ_*MBP*_ is an incremental time step. The assessment of the optimal values of *n* and the scale Δ_*MBP*_ is based on the statistical analysis of residence times detailed below. The role of *MBP*_*n*_ in the construction of CRTs is illustrated in S2 Fig.

#### Identification of the optimal timescale for the construction of continuous residence times

The acoustic data is processed according to incremental values of *MBP*_*n*_ defined in Eq (1), with *n* in the interval [1 : *N*], thus leading to *N* sets of continuous residence times. The choice of *N* determines the larger timescale *MBP*_*N*_ and must be high enough to encompass the timescale of interest. As a second step, for each *n*, the survival curves of residence times *S*_*MBP*_*n*__(*t*) are constructed by taking the fraction of CRT that are larger than *t*. The approaches of survival analysis have been firstly applied to medical research, where the term “survival” was directly related to the lifetime of an individual. In this paper we exploited the same approaches by considering “residence times” in the place of “lifetimes” and extending the concept of “survival” to the fish “residency” at a given receiver. Generally speaking, survival curves *S*(*t*) inform on the probability of a failure event to occur at a certain time *t* [33]. Here, the failure event corresponds to the interruption of a continuous residence time, either due to the absence of the acoustic signal for periods larger than *MBP*_*n*_ or to the detection of fish at another receiver. The assessment of the optimal *MBP*_*n*_ (denoted below as $MBP_{n}^{*}$) is based on the comparison of the set of survival curves of residence times obtained at different *n*. Indeed, random (noise) or behavioral events in the raw data lead to different survival curves of residence times for different choices of *n* below a certain unknown threshold. Starting from a low *n* and increasing its value, our guess is that survival curves of residence times should stabilize after a given threshold n* associated to the time scale $MBP_{n}^{*}$ at which those events do not affect the estimate of residence times any more. In this view, the statistical comparison of residence times obtained at variable *MBP*_*n*_ can reveal the underlying pattern of acoustic detections and the related behavioral processes. The comparison relies on the calculation of the sum of squared residuals (SSR) among pairs of survival curves of residence times. For each *n*, we considered pairs of survival curves (*S*_*MBP*_*n*__(*t*), *S*_*MBP*_*n*_+Δ_*MBP*__(*t*)) associated to incremental values of the variable *MBP*_*n*_ (see Eq (1)). Since different *n* imply different CRT for the same raw dataset (see S2 Fig), the survival curves of residence times were first resampled over a series of regular time steps Δ*t* by performing a linear interpolation. The linear interpolation was conducted each time the distance between two subsequent points (CRT) of the survival curve fell below a given threshold *t*_*max*_. Conversely, each time two points were separated by a distance larger than *t*_*max*_, the interpolation was not performed and this part of the survival curve was consequently discarded. Finally, for each pair of interpolated survival curves (*S*_*MBP*_*n*__(Δ*t*), *S*_*MBP*_*n*_+Δ_*MBP*__(Δ*t*)), the SSR was estimated and then renormalized (denoted below as rSSR), by dividing by the total number of data points T considered in its calculation, leading to:

$$rSSR\left(MBP_{n}\right)=\frac{\sum_{i=1}^{T}{\left[S_{MBP_{n}}\left(\Delta t_{i}\right)-S_{MBP_{n}+\Delta_{MBP}}\left(\Delta t_{i}\right)\right]}^{2}}{T}$$ (2)

where the index *i* runs over all timesteps Δ*t*_*i*_ where both the interpolated survival curves are defined. The criterion defined in Eq (2) ensured a consistent comparison among pairs of curves obtained at different *MBP*_*n*_. Finally, the convergence of the survival curves of residence times was assessed from the plots of rSSR(*MBP*_*n*_) as a function of *MBP*_*n*_.

### Method validation and applications

#### Validation of the method through simulations

Our approach was tested over a simulated sample of acoustic detections obtained from the behavioral model introduced in [34, 35]. In its simplest non-social formulation, this model describes the dynamics of a set of *N*_*F*_ independent fish within an array of *p* receivers, through a system of *p* differential equations of the form:

$$\frac{dX_{i}}{dt}=\mu X_{u}-\theta X_{i}$$ (3)

where *X*_*i*_ is the number of individual fish present at receiver *i* at time *t*, *X*_*u*_ is the number of fish that are present out of the receivers, such that $N_{F}=\sum_{i=1}^{p}X_{i}+X_{u}$ and *μ* and *θ* express the probability to join or leave a receiver, respectively. As far as a fish is present at a receiver it is considered within its detection range and is thus detectable at each time step. Based on Eq (3), we simulated different patterns of acoustic detections following the three model scenarios described below.

#### Scenario 1. Single exponential model with noise

The first scenario is a memory-less dynamic example, where the probabilities of joining/leaving a receiver do not depend on the time spent outside/at the receiver. As such, *μ* and *θ* in Eq (3) are two time-independent constants. The timescale associated to the residence times recorded at the same receiver is related to the inverse probability to leave the receiver 1/*θ* and the survival curves of residence times follow *S*(*t*) = exp(−*θt*) [34]. Similarly, absence times are governed by the timescale 1/*μ* and follow the exponential survival curve *S*(*t*) = exp(−*μt*). Here, in addition to the behavioral events described by Eq (3), we considered a second timescale 1/*η* related to the effects of environmental/instrumental noise. Each time an individual was present at a receiver, an acoustic detection was recorded with probability *η*, with *η* ≤ 1 (*η* = 1 implying absence of noise). The parameter *η* was taken as a constant, i.e. the noise events were independent of the time spent by the fish at the receiver. In the following, the parameters *μ* and *θ* were chosen much smaller than *η*, supposing the existence of two distinct timescales respectively related to fish behavior and external noise, with the former associated to larger timescales.

#### Scenario 2. Time-dependent sigmoidal model

Within the second scenario the probability to join the receiver increases with the time spent out of it and corresponds to a sigmoid function of the form:

$$\mu \left(\tau \right)=\frac{\mu_{\infty }}{1+K\exp(-\gamma t)}$$ (4)

where *τ* is the time spent by an individual outside the receivers, *μ*_∞_ is the asymptotic probability to reach the receiver at large times and *K* and *γ* are two constants. In this case Eq (3) becomes:

$$\frac{dX_{i}}{dt}=\int_{0}^{t}\mu \left(\tau \right)X_{u}\left(t,\tau \right)d\tau -\theta X_{i}$$ (5)

where *X*_*u*_(*t*, *τ*) represents the number of fish which have spent a time *τ* outside the receivers at time *t*. Oppositely, the timescale associated to the residence times recorded at the same receiver is kept constant and equals 1/*θ* like in Scenario 1. As *γ* increases, Eq (4) approaches a step-like function, switching from small to large values around τ* = ln(*K*)/*γ*. In this limiting case, Eq (4) involves two main timescales for absence times, 1/*μ*_∞_ and (1+*K*)/*μ*_∞_.

#### Scenario 3: Time-dependent sigmoidal model with noise

The third scenario is a mixture of the two above, where the probability to reach the receivers is time dependent (Eq (4), Scenario 2) and environmental/instrumental noise affects the acoustic records with probability *η* (Scenario 1).

For all scenarios, the simulated acoustic detections were recorded following a Monte Carlo algorithm over a run of 100.000 time steps [34], following Eqs (3 and 4) for *p* = 2 receivers and *N*_*F*_ = 1000 fish individuals. The model parameters specific to each scenario are reported in Table 1. The simulated set of acoustic detections were processed at different *MBP*_*n*_ ranging between 100 and 2000 time steps, with Δ_*MBP*_ = 100 (Eq (1)). In addition to this set of *MBP*_*n*_, the survival curves were compared with those obtained for *MBP*_0_ = 1, i.e., a timescale corresponding to the timestep of the simulation. The linear interpolation employed in the calculation of the rSSR (Eq (2)) was conducted with Δ*t* = 1 and *t*_*max*_ = 100.

**Table 1:** Model parameters for the three scenarios. Columns from left to right indicate the parameters related to the probability to depart from a receiver, the probability to reach a receiver and the probability of detecting a fish due to environmental/instrumental noise.

Scenario	Prob. to depart	Prob. to reach	Noise
1	*θ* = 0.0002	*μ* = 0.0001	*η* = 1, 0.1, 0.01, 0.005
2	*θ* = 0.02	*μ*_∞_ = 0.01	-
		*K* = 1000	
		*γ* = 0.01, 0.02, 0.04, 0.08	
3	*θ* = 0.0002	*μ*_∞_ = 0.01	*η* = 0.1
		*K* = 1000	
		*γ* = 0.01	

### Case study datasets

We applied our methodological framework to two datasets, concerning two different species and acoustic array characteristics: (i) data collected on 37 bigeye scads (*Selar crumeophthalmus*) in an array of 9 acoustic receivers in Reunion Island (South Western Indian Ocean) in June-July 2006 [18, 27] and (ii) data collected on 32 yellowfin tuna (*Thunnus albacares*) in an array of 13 acoustic receivers located around the island of Oahu, Hawaii (Central Pacific Ocean) in February-August 2003 [17, 26]. Details on the two datasets can be found in Table 2 and in the Supporting Information (S3 Fig and S1 Text).

For the first application of the method, which was focused on small timescales, both datasets were processed with values of *MBP*_*n*_ ranging between 10 and 120 min, following Eq (1) with Δ_*MBP*_ = 10 min. The choice of 10 min corresponded to the minimum time interval required for the detection of two consecutive emissions suggested by the constructor (www.vemco.com). For larger timescales, survival curves of residence times were constructed using values of *MBP*_*n*_ between 2 and 48 h obtained for Δ_*MBP*_ = 2 h in Eq (1). The linear interpolation employed in the calculation of the rSSR (Eq (2)) was conducted with Δ*t* = 10 min and *t*_*max*_ = 4 h.

**Table 2:** Experimental data. Columns from left to right: species, number of tagged individuals, number of instrumented FADs, location and acoustic telemetry equipment (receiver and tag type) for the two datasets used in this study.

Species	Tag IDs	FAD IDs	Location	Date	Equipment
*Selar chrumeophthalmus*	37	9	Reunion Island	2006 (June-July)	VEMCO VR2/V7
*Thunnus albacares*	32	13	Oahu, Hawaii	2003 (February-May)	VEMCO VR2/V16

#### Ethics Statement

The fish experimental protocols for the field studies conducted in Réunion Island were permitted under the Aquarium of Reunion Island animal care certificates delivered by the French Veterinary Medicine Directorate. Protocols were carried out with the authority of the National Veterinary School of Nantes (France) validating a certificate of training in animal experimentation and a degree in experimental surgery on fish. The fish experimental protocols for the field studies conducted in Hawaii were specifically approved by the University of Hawaii Institutional Animal Care and Use Committee (IACUC).

## Results

### Validation of the method through a simulated set of acoustic detections

**Fig 1:**
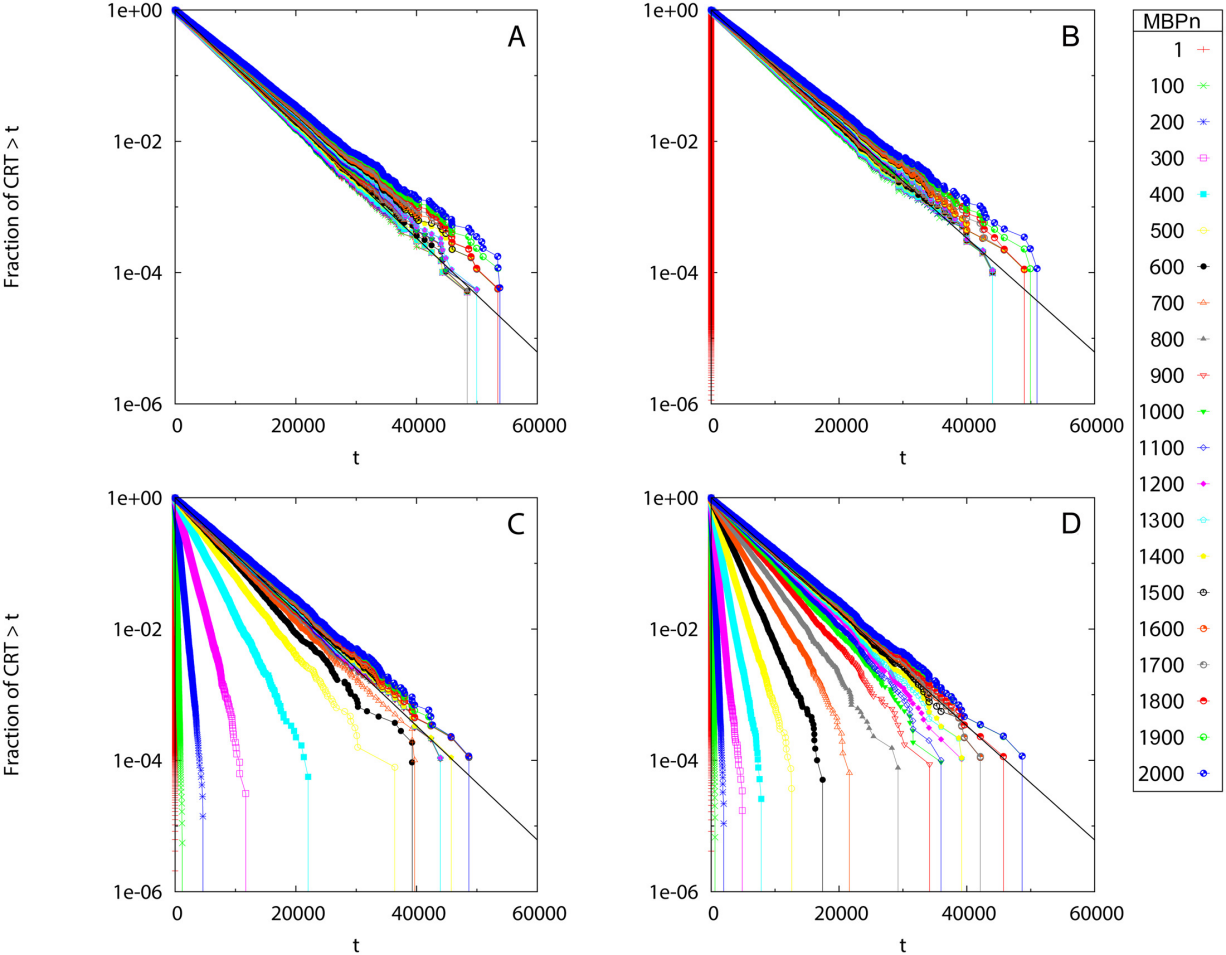
Scenario 1: Survival curves of CRTs. The survival curves are obtained for different values of *MBP*_*n*_ (see legend) and different noise parameters: *η* = 1 (A), 0.1 (B), 0.01 (C) and 0.005 (D). The *y* axis is in logarithmic scale. Black line: the theoretical survival curve of residence times *S*(*t*) = exp(−0.0002*t*).

**Fig 2:**
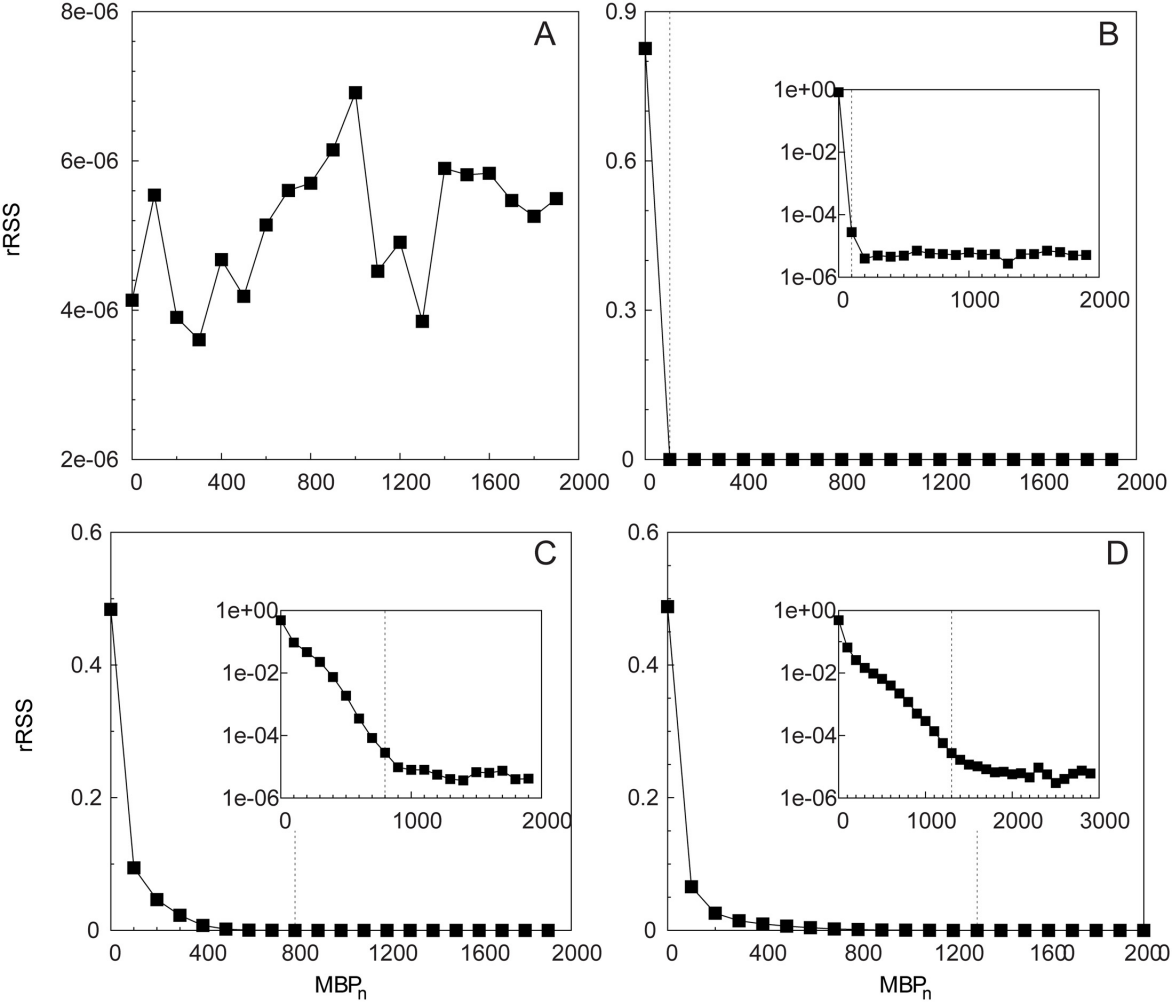
Scenario 1: Renormalized sum of squared residuals. The rSSR is calculated among pairs of survival curves of residence times with Δ_*MBP*_ = 100 and different values of the noise parameter: *η* = 1 (A), 0.1 (B), 0.01 (C) and 0.005 (D). The vertical dashed line represent the MBP value at which the survival curve of residence times mostly approached the theoretical curve. Insets: the same in semi-logarithmic scale.

Fig 1 shows the survival curves of residence times for Scenario 1 obtained for the simulated set of acoustic detections processed at different *MBP*_*n*_. The linearity of the curves in semi-logarithmic scale (apart from the large times deviation due to the finite simulation time) demonstrated that all curves followed an exponential law, as expected from model construction. The survival curves of residence times showed a different variability with respect to the MBP choice depending on the value of the noise parameter *η*. For *η* = 1 (no noise) there was little dependence on *MBP*_*n*_, whereas for *η* = 0.1, 0.01 and 0.005 the curves tended to converge only above a non-zero value of *MBP*_*n*_ which increased with decreasing *η* (i.e., for increasing noise). The estimated rSSR reported in Fig 2 assessed this convergence more quantitatively. For *η* = 1 (Fig 2A) the rSSR fluctuated around a constant value close to zero. Oppositely, a decreasing trend in the rSSR was evident for *η* < 1, where the rSSR stabilized after $MBP_{n}^{*}=100$ (*η* = 0.1, Fig 2B), $MBP_{n}^{*}$ = 900 (*η* = 0.01, Fig 2C) and $MBP_{n}^{*}$ = 1500 (*η* = 0.005, Fig 2D). Remarkably, at these values of $MBP_{n}^{*}$ the survival curves of residence times approached the theoretical form *S*(*t*) = exp(−*θt*) which demonstrated the validity of our approach. The case *η* = 0.005 (Fig 2D) showed a smoother decrease in the rSSR around $MBP_{n}^{*}$ rather than the sharp decrease to the convergence point found for higher *η* values. The sensitivity of our method with respect to the choice of Δ_*MBP*_ was tested in S4 Fig. The convergence of the rSSR did not depend on the choice of Δ_*MBP*_ but the identification of MBP* was looser for higher Δ_*MBP*_ values.

**Fig 3:**
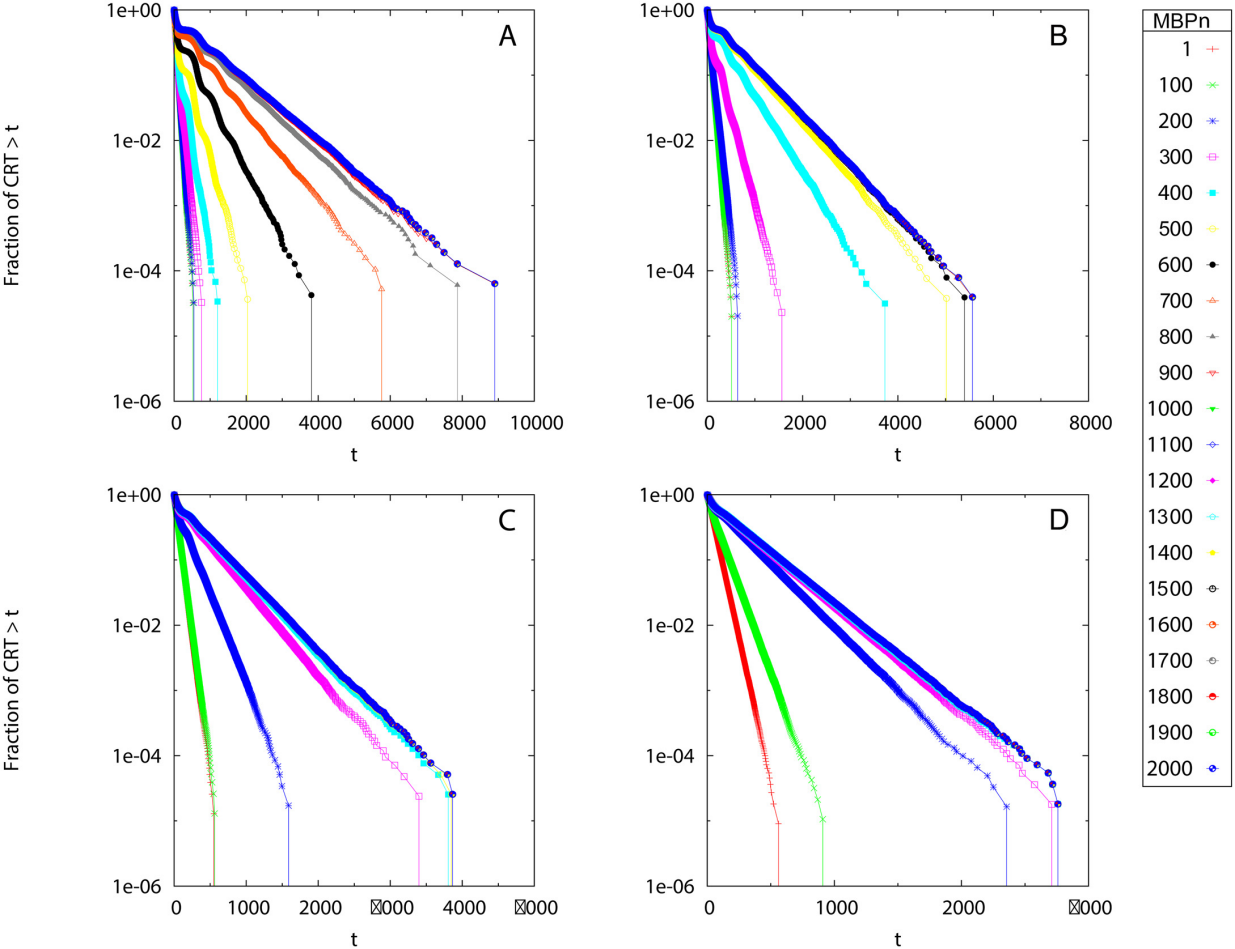
Scenario 2: Survival curves of CRTs. The survival curves are obtained for different values of *MBP*_*n*_ (see legend) and different model parameters in Eq (4): *γ* = 0.01 (A), 0.02 (B), 0.04 (C) and 0.08 (D). The *y* axis is in logarithmic scale.

**Fig 4:**
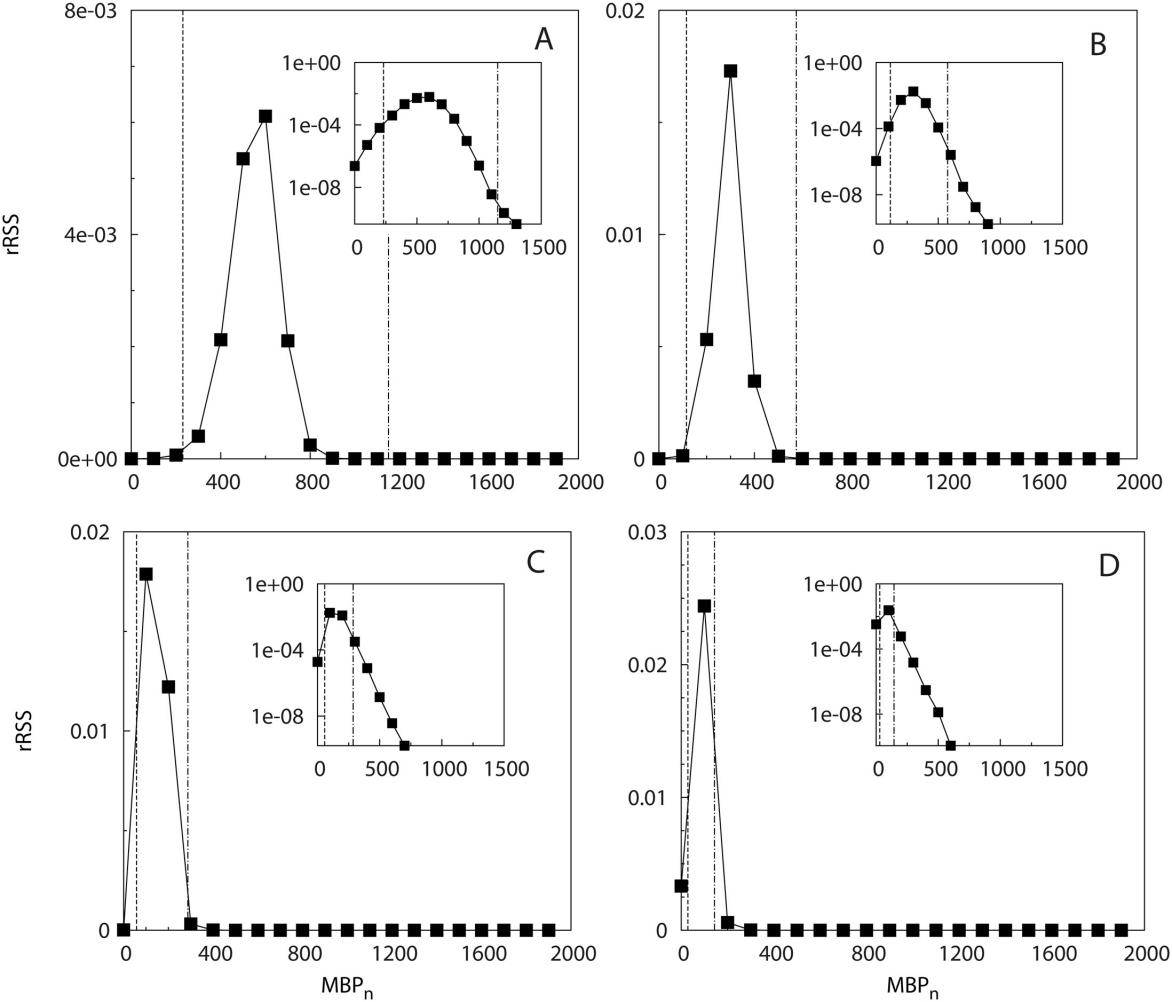
Scenario 2: Renormalized sum of squared residuals. The rSSR is calculated among pairs of survival curves of residence times with Δ_*MBP*_ = 100 for different model parameters in Eq (4): *γ* = 0.01 (A), 0.02 (B), 0.04 (C) and 0.08 (D). The vertical lines represent the time values *t* where *μ*(*t*) = 1%*μ*_∞_ (dashed line) and *μ*(*t*) = 99%*μ*_∞_ (dot-dashed line). Inset: the same in semi-logarithmic scale.

In contrast with Scenario 1, the survival curves of residence times *S*(*t*) for Scenario 2 presented a varying slope as a function of time, with characteristic plateaus at small *t*, see Fig 3. From visual inspection, two groups of homogeneity classes at small and large *MBP*_*n*_ appeared. The calculation of the rSSR (Fig 4) revealed the presence of two timescales ($MBP_{1}^{*}$ and $MBP_{2}^{*}$) below/above which the curves showed small changes (i.e., small rSSR). The values of $MBP_{1}^{*}$ and $MBP_{2}^{*}$ depended on the model parameter *γ* (see Eq (4)) and decreased when increasing *γ*. The range of *MBP*_*n*_ where the CRTs showed a higher variability (i.e., larger rSSR) were consistent with the theoretical timescales where the probability to reach the receivers defined in Eq (4) moves from small values (*μ*(*t*) = 1% *μ*_∞_) to the asymptotic limit (*μ*(*t*) = 99% *μ*_∞_) (see vertical lines in Fig 4), which demonstrates the consistency of our approach.

**Fig 5:**
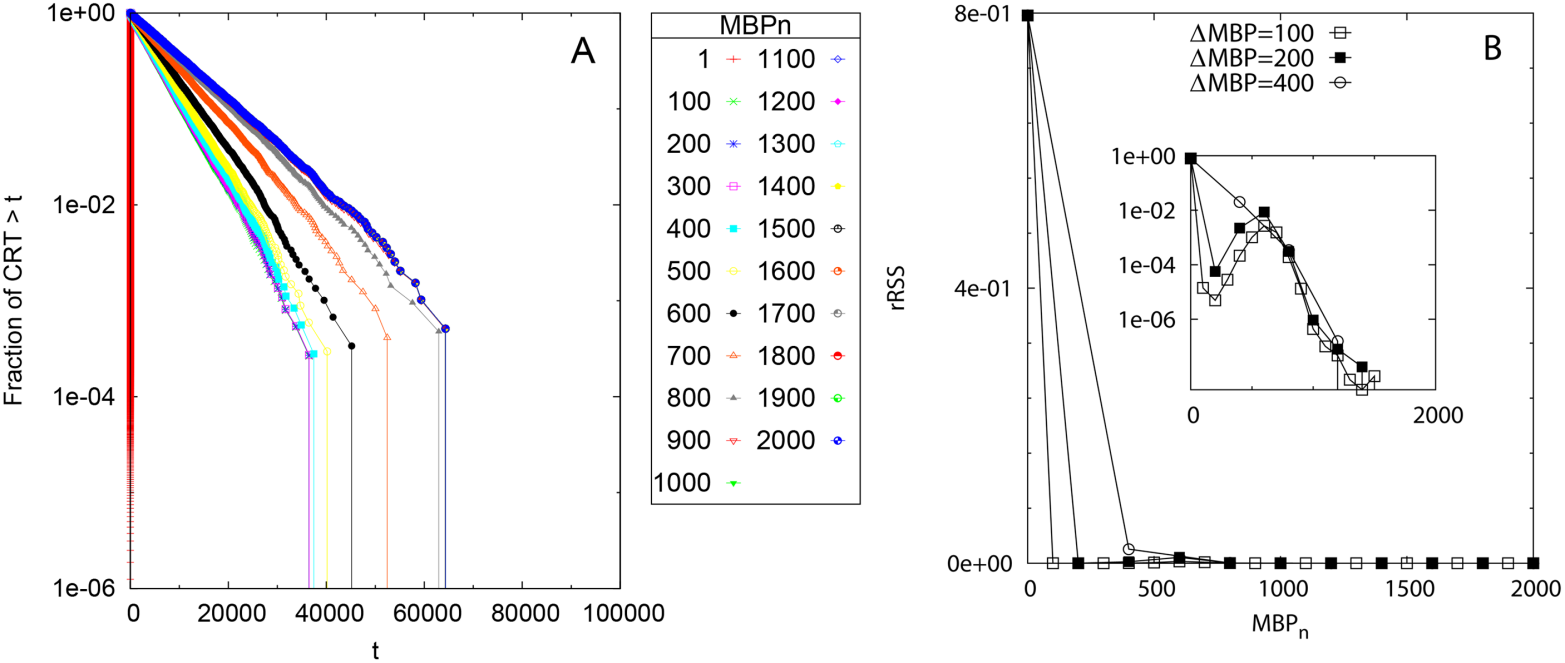
Scenario 3: Survival curves of CRT and renormalized sum of squared residuals. (A) Survival curves of CRT obtained for different values of *MBP*_*n*_ (see legend). (B) rSSR in semi-logarithmic scale calculated among pairs of survival curves of residence times with variable Δ_*MBP*_ (see legend). Inset: the same in semi-logarithmic scale.

Fig 5 shows the application of our approach for the mixed case where both multiple behavioral timescales and environmental/instrumental noise coexist (Scenario 3). The visual inspection of survival curves of residence times (Fig 5A) showed a clear difference between the curve obtained for *MBP*_0_ = 1 and larger *MBP*_*n*_, similarly to what was found for Scenario 1. Moreover, it was possible to visually identify two homogeneity classes at larger *MBP*_*n*_, similarly to what was found for Scenario 2. The behavior of the rSSR was consistent with these findings and showed a clear jump to small values for *MBP*_*n*_ > *MBP*_0_. For Δ_*MBP*_ = 100 the rSSR was non-monotonous and demonstrated a first convergence to small values for 100 ≤ *MBP*_*n*_ ≤ 400 and a second range of convergence after $MBP_{n}^{*}=1000$ (see inset of Fig 5B). When increasing Δ_*MBP*_ up to 400, the first zone of convergence at small *MBP*_*n*_ was no more observable whereas the larger timescale $MBP_{n}^{*}=1000$, beyond which the rSSR was equal to zero, was consistently identified.

### Application to realistic acoustic datasets

#### First application: Continuous residence times to overcome environmental/instrumental noise

**Fig 6:**
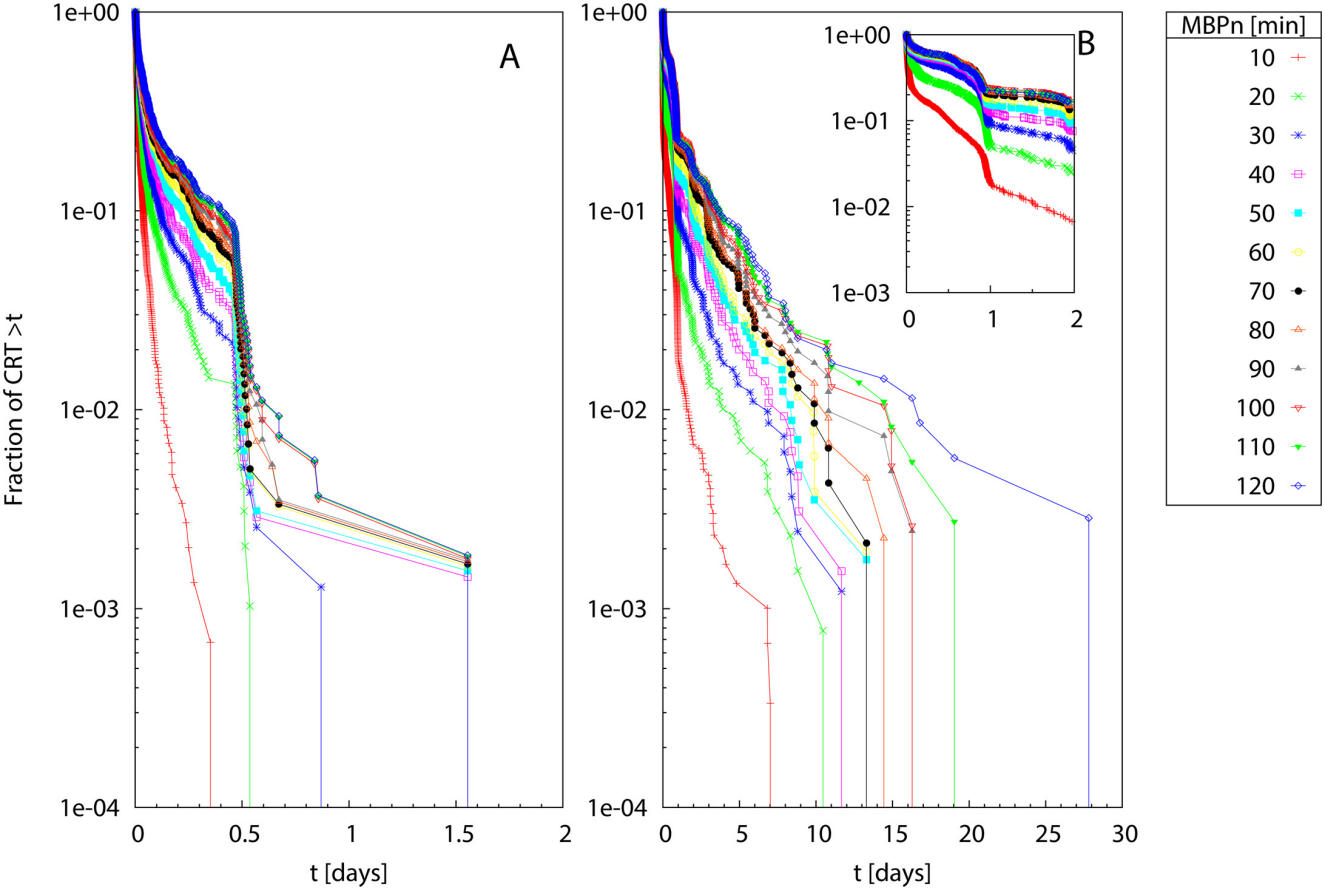
Field data: Survival curves of CRTs at small timescales. Survival curves of CRTs obtained for MBP ranging between 10 up to 120 min by intervals of 10 min (see legend) in semi-logarithmic scale for (A) bigeye scad (B) yellowfin tuna.

**Fig 7:**
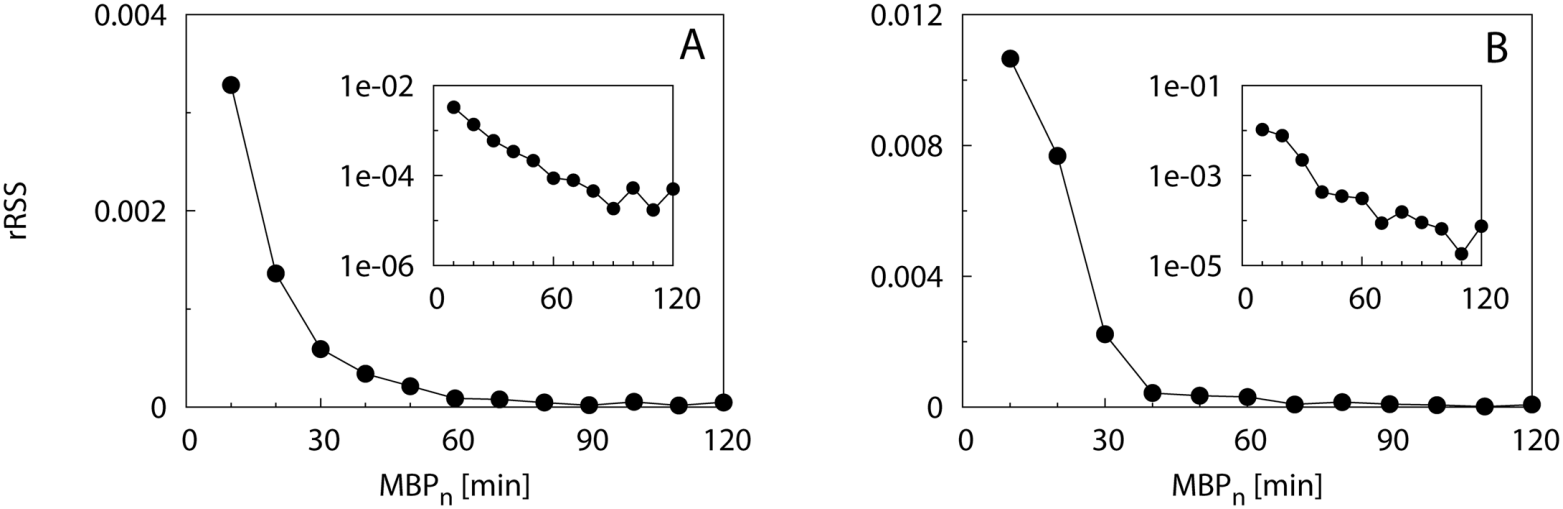
Field data: Renormalized sum of squared residuals at small timescales. The rSSR is calculated among pairs of survival curves (*S*(*t*_*MBP*_*n*__), *S*(*t*_*MBP*_*n*_+Δ_*MBP*__)) with Δ_*MBP*_ = 10 min for bigeye scad (A) and yellowfin tuna (B). Insets: the same in semi-logarithmic scale.

Fig 6 shows the survival curves of CRTs for bigeye scad (Fig 6A) and yellowfin tuna (Fig 6B) obtained for increasing *MBP*_*n*_ with Δ_*MBP*_ = 10 min. The survival curves presented multiple slopes and their shape varied according to the species and values of *MBP*_*n*_, with yellowfin tuna presenting a larger range of residence times. However, a gradual convergence of residence times when increasing *MBP*_*n*_ emerged when inspecting the survival curves at short timescales (< 2 days, see inset of Fig 6B for yellowfin tuna). The estimated rSSR in Fig 7 decreased less rapidly after MBP* = 60 min, where it stabilized to values close to zero for both species (see insets of Fig 7A and 7B). The sensitivity analysis conducted for larger Δ_*MBP*_ (20 min and 30 min) confirmed this result (S5 Fig).

#### Second application: Continuous residence times to overcome absences of signal related to diel excursions

**Fig 8:**
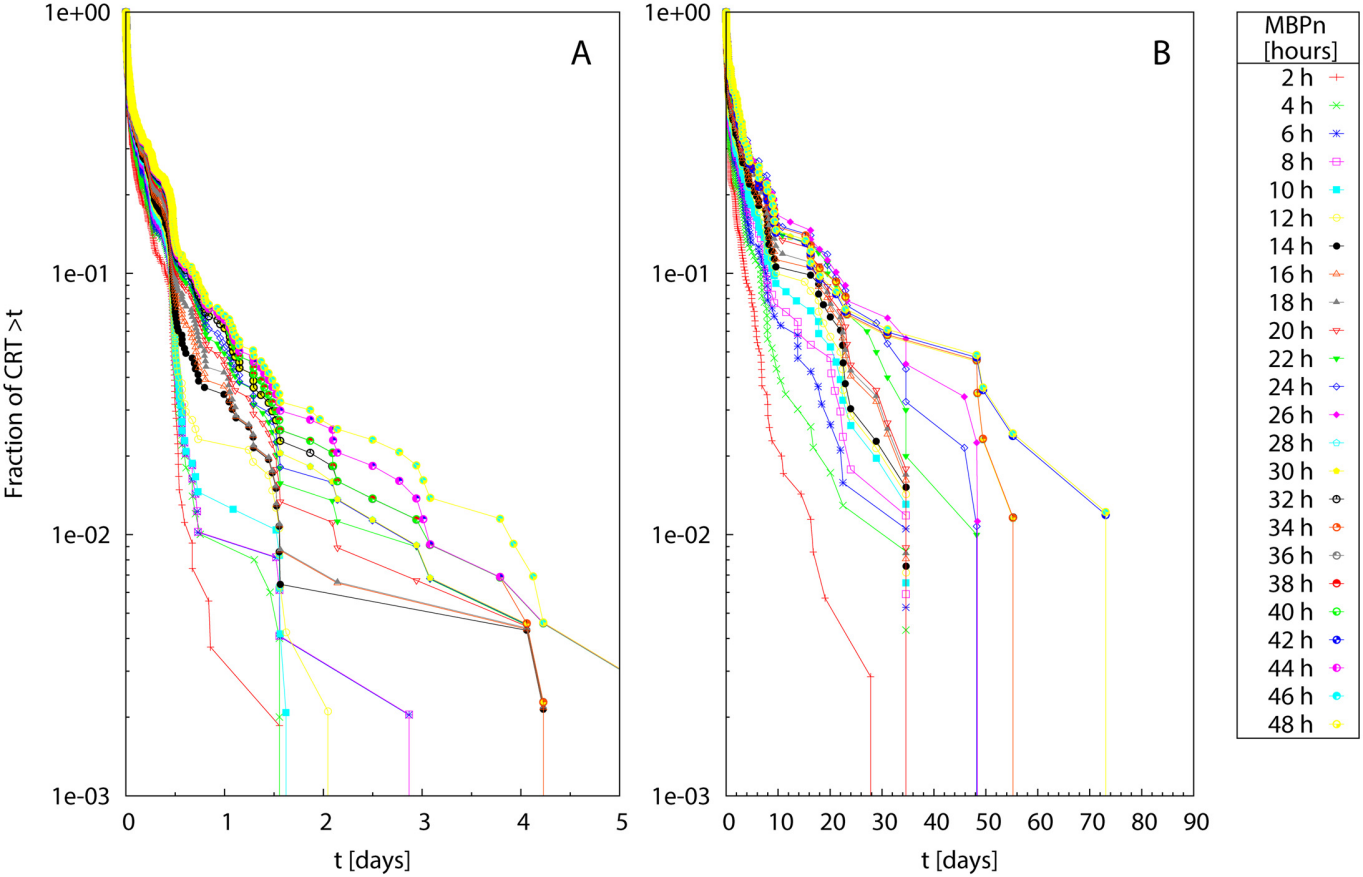
Field data: Survival curves of CRTs at large timescales. Survival curves calculated for *MBP*_*n*_ ranging between 2 h and 48 h (see caption) for (A) Bigeye scad (B) yellowfin tuna.

**Fig 9:**
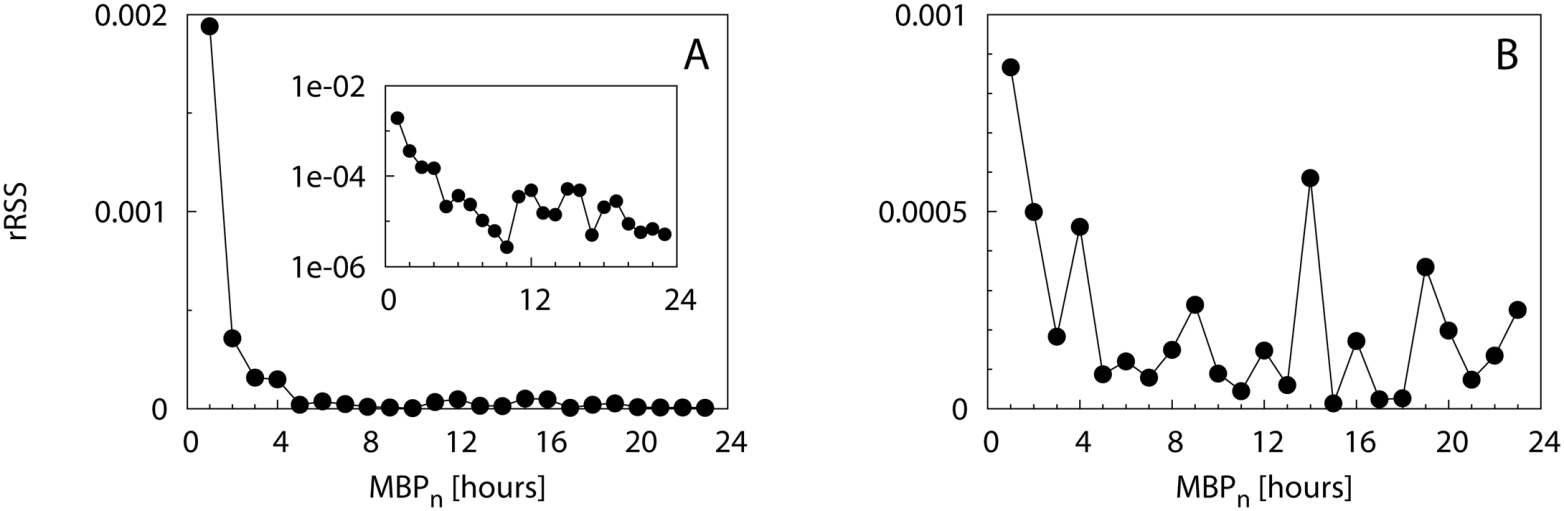
Field data: Renormalized sum of squared residuals at large timescales. The rSSR is calculated over pairs of survival curves with Δ_*MBP*_ = 2 h (stars), 4 h (empty squares), 6 h (filled squares) and 8 h (empty circles) for bigeye scad (A) and yellowfin tuna (B). Insets: the same in semi-logarithmic scale.

Fig 8 shows the survival curves of residence times obtained for Δ_*MBP*_ = 2 h. Even for large values of *MBP*_*n*_, the residence times of bigeye scad (Fig 8A) were quite short (on the order of few consecutive days) when compared to those of yellowfin tuna (Fig 8B), which could remain associated with the same receiver for several weeks or months. Again, survival curves showed varying shapes depending on *n*, signaling a clear sensitivity of the residence times to the MBP choice. Their convergence was quantified in Fig 9, where the rSSR was calculated for Δ_*MBP*_ = 2, 4, 6 and 8 h. For Δ_*MBP*_ = 4, 6 and 8 h the rSSR approached constant values around MBP* = 24 h whereas, for Δ_*MBP*_ = 2 h the rSSR attained a constant value at earlier MBP values, around MBP* = 6 h, for both bigeye scad and yellowfin tuna.

## Discussion

The processing and analysis of data coming from acoustic tagged animals detected at specific points in space requires care. Without a robust methodological approach capable of assessing the optimal timescales for processing the acoustic data, the risk of misinterpretation could be high. The quantitative approach proposed herein allows identifying these timescales and assessing their range of validity, based on the statistical comparison of survival curves of residence times. The starting point of our approach is the construction of continuous residence times (CRT) from discrete acoustic detections based on a key variable, the Maximum Blanking Period, MBP (e.g. [27]) This variable, related to the maximum time that is allowed between two subsequent acoustic detections for considering that a tagged animal is still present, determines when discrete detections turn into CRTs. The core of the approach relies on the fact that survival curves of residence times obtained with subsequent values of MBP tend to converge when overcoming underlying patterns in the signal related to environmental/instrumental noise and/or behavior of the tagged animals. Therefore, the comparison of survival curves leads to the natural emergence of this temporal scale and its range of validity, as a direct output of the data analysis. This methodological framework contrasts with subjective (“intuitive”) choices (whose range of validity is unknown) that can be found in the literature.

The validity of the method and its ability to accurately identify temporal patterns in the raw data was demonstrated through simulations. Contrary to what is generally found in the literature of survival analysis, the comparison of survival curves here involved the same data (i.e., the acoustic detections at the different receivers) processed in different ways according to the different values of the MBP. Therefore, the hypothesis of comparing two independent datasets does not hold in this case. Our results showed that the convergence of the renormalized sum of squared residuals (rSSR) provided an effective criterion for revealing the optimal MBP values that overcame the effects of external noise. To the purpose of validating our method, the model was first constructed by testing the ideal situation of two well-separated timescales related to the noise and the behavioral excursions (Scenario 1). To this purpose, a time-independent exponential model was employed, inspired by the recent theoretical and experimental findings on the associative behavior of tropical tuna around FADs ([26, 34]). The rSSR showed a sharp decrease and an early stabilization at the optimal MPB value. On the other hand, we revealed a smoother transition and a slight overestimation of the optimal MBP when the two timescales approached each other, a situation which makes the identification of the optimal MBP more difficult. Secondly, the method was tested on a sigmoidal model associated with a time-dependent behavior out of the receiver (Scenario 2). This model leads to a distribution of absence times close to a Gaussian distribution, a situation that can be found in many biological systems. In this case our approach could successfully identify two sets of homogeneous survival curves, located below/above the characteristic timescale (τ*) where Eq (4) switches from small to high values. Finally, the method was tested in the presence of multiple behavioral timescales and noise (Scenario 3), a situation which is the most common in field experiments. Our approach could identify an appropriate timescale for constructing residence times without the effects of noise. At the same time it could be employed at a larger timescale for the identification of residence times that overcame the short-term behavioral excursions.

The application of our method to the bigeye scad and yellowfin tuna acoustic data demonstrated that realistic datasets present a multiplicity of timescales, similarly to model Scenario 3. In the first application we illustrated how our methodological framework allows identifying the smallest timescale that can support a fine-scale analysis of animal behavior, free of the bias due to environmental and instrumental noise. Both our case studies presented very pronounced differences in the survival curve for small values of the MBP (10, 20, 30 min) whereas beyond 60 min survival curves tended to converge (Fig 7). Similarly to Scenario 1 (case *η* = 0.005, Fig 2D), the rSSR showed a smooth convergence after MBP* = 60 min for both species, rather than a sharp jump to constant values. On the other hand, the small fluctuations of the rSSR found for *MBP* > MBP* (Fig 7), as well as the characteristic plateaus observable in the slopes of the survival curves (Fig 6), indicate the closeness of other timescales in the raw data, similarly to Scenario 2 (e.g. Fig 3A). This result ensures that the widespread choice of MBP = 60 min, used in the past literature ([18, 31]) for the analysis of small-scale behavior for these two species was high enough to avoid the possible biases related to environmental/instrumental noise.

The second application illustrated that our methodological framework can be used to identify the presence of multiple behavioral timescales in the pattern of acoustic detections. A timescale of 24 h naturally emerged from the comparison of survival curves at increasing *MBP*_*n*_ for both species. This result, which ensures the validity of the widespread choice of MBP = 24 h for tunas [16, 17, 31], used in the past literature to study site fidelity, can be interpreted in the light of the different excursions taking place when the two fish species are associated with FADs. For tuna, it is well known that the association radius around the FAD can extend to several hundred meters [36], leading to long absences of the signal when a fish is outside the range of detection. These excursions are likely to be linked with feeding events away from the FADs [22–25]. On the other hand, the large MBP* found for bigeye scad was quite surprising since this species is known to stay very close to the FAD. However, previous studies demonstrated the presence of diel night excursions of bigeye scads out of the FADs [18]. Moreover, possible current effects could induce fish to occupy positions further from the FAD (e.g. upstream to the current, see [37]), inducing in/out excursions out of the detection range of the receiver similarly to tuna.

Sensitivity analysis indicated that the detection of the optimal MBP might be sensitive to the chosen increment Δ_*MBP*_. If the difference between two consecutive MBP is too high, the identification of the appropriate time scale is looser. This situation was encountered in S4 Fig for Scenario 1. The opposite situation was found for field data (Fig 9), where an earlier convergence of survival curves around MBP* = 6 h was found for small increments Δ_*MBP*_ = 2 h. This result can be explained in the light of the multiplicity of timescales involved in the acoustic data. The increment Δ_*MBP*_ = 2 h allows detecting the variations among survival curves related to small excursions around the receivers but is not large enough to account for the full range of timescales involved in the diel behavior of fish.

Alternative approaches to tackle the problem of detection probability and efficiency are proposed in the capture-recapture and trapping literature. Notably, these methods are either based on the explicit definition of home range in the capture function [38] or they exploit the link between environmental covariates and variation in the detection range [39]. Applied to passive acoustic telemetry, home ranges would correspond to the range of detection of an acoustic receiver. However, the assumptions on the stationary distribution of the home range in two dimensions [40] do not hold for realistic passive acoustic datasets. Similarly, the data required in the second class of approaches [39] are difficult to monitor within realistic experiments, that are often conducted in remote areas. Moreover, precisely quantifying the effects of all factors (e.g., tag characteristics, species characteristics, such as horizontal and vertical behavior, environmental noise) involved in detection-range variability is hardly possible [6, 14, 41, 42]. On the contrary, we based our approach on the convergence of survival curves of residence times beyond a given timescale, without accounting explicitly for the origin of signal absence. The same principles can be applied to a large variety of datasets like those employed in the capture-mark-recapture and mark-resight literature [43], where discrete records of animal detections are translated to continuous variables related to the estimated density of a species in time and space.

### Conclusion

Our method could be successfully applied for estimating residence times both at small and larger timescales, highlighting that this methodological framework is general enough to be employed for different scientific objectives, species and experimental settings. Applications of passive acoustic telemetry are quite broad, from fundamental studies of animal behavior and spatio-temporal pattern to conservation objectives such as the assessment of marine reserve efficiency and habitat use [1, 6, 9]. In particular, the exact proportion of time animals spend closely associated to a receiver (e.g corresponding to habitat types) within a day or over the entire experiment (site fidelity) are important behavioral metrics that need to be assessed for management purpose. Quantification of those metrics cannot be left to the authors’ intuition and needs to be performed using dedicated approaches that can identify the most appropriate temporal scale for the acoustic data processing, as proposed in this study. Given the high cost of tagging and field work campaigns, optimizing the quantity –and quality—of data extracted from this kind of experiments and allowing comparison among different datasets represents a significant contribution to the field.

## Supporting Information

S1 Text Dataset Description. (PDF)

S1 File Acoustic detections recorded in Reunion Island from tagged individuals of bigeye scad. Each line corresponds to an acoustic detection. Columns from left to right refer respectively to the FAD ID, Fish ID, incremental datetime expressed in units of seconds and date (formatted as DD MM YYYY h min sec).
(TXT)

S2 File Acoustic detections recorded in Hawaii from tagged individuals of yellowfin tuna. Each line corresponds to an acoustic detection. Columns from left to right refer respectively to the FAD ID, Fish ID, incremental datetime expressed in units of seconds and date (formatted as DD MM YYYY h min sec).
(TXT)

S1 Fig Snapshot of the acoustic detections (crosses) recorded by an acoustic receiver for different tagged fish (Tag ID). Data from [18, 27].
(EPS)

S2 Fig Schematic representation of the construction of CRTs from discrete passive acoustic detections and different *MBP*_*n*_ choices. Each cell of the array corresponds to a time unit (tu). The two symbols (blue and red crosses) correspond to the detections at two different receivers. The data is processed with increasing *MBP*_*n*_ following Eq (1) and taking Δ_*MBP*_ = 1 tu. Raw data (A); Processed data with *MBP*_1_ = 1 tu (B); *MBP*_2_ = 2 tu (C) and *MBP*_2_ = 3 tu (D).
(PNG)

S3 Fig Location of the FAD arrays for the two experimental datasets (A) in Reunion Island (B) in Hawaii. Each equipped FAD is represented by a black dot.
(PNG)

S4 Fig Renormalized sum of squared residuals. RSS in semi-logarithmic scale calculated among pairs of survival curves for the model Scenario 1, with noise parameter *η* = 0.01 and different values of Δ_*MBP*_ (see legend).
(EPS)

S5 Fig Renormalized sum of squared residuals. RSS calculated among pairs of survival curves following Eq (2) with Δ_*MBP*_ = 10 min (empty squares), 20 min (filled squares) and 30 min (empty circles) for bigeye scad (A) and yellowfin tuna (B). Insets: the same in semi-logarithmic scale.
(EPS)
