# Supplementary material for: A Methodological Framework to Estimate the Site Fidelity of Tagged Animals Using Passive Acoustic Telemetry
Source: PLoS One. 2015 Aug 11;10(8):e0134002. doi: 10.1371/journal.pone.0134002 (PMC4532465; doi:10.1371/journal.pone.0134002)
Supplement: S1 Text — (PDF) [file pone.0134002.s001.pdf]

S1 Text

Dataset Description

In both experiments, all FADs were equipped with VEMCO VR2 sonic receivers designed to detect a wide range of uniquely coded sonic transmitter tags. These receivers were mounted directly to the FAD mooring system approximately 18 m below the sea surface. The acoustic tags VEMCO V7 (69 kHz, V7-2L-R04K, 70 – 140 s delay, rated battery life 130–150 days) for bigeye scads and V16 (69 kHz, V16-4H-R256, 5–30 s delay, rated battery life 344 days) for yellowfin tuna were inserted in the peritoneal cavity of fish using standard fish tag implantation techniques. All fish were tagged during daytime at the vicinity of a FAD. Each time a tag (in a fish) transmitted within the range of detection of an acoustic receiver (experimentally estimated to 150 m in La Reunion Island and 600-1100 m in Hawaii), the acoustic receiver recorded the unique tag ID and the time of reception. Further details on the experimental settings, tagging procedures and individual characteristics can be found in [27] and [17], respectively. The two FAD-network arrays are shown in S3 Fig. The two raw data files containing the acoustic detections can be found in the Supporting information files S1 File and S2 File.
