# Supplementary figures and images for: A Methodological Framework to Estimate the Site Fidelity of Tagged Animals Using Passive Acoustic Telemetry
Source: PLoS One. 2015 Aug 11;10(8):e0134002. doi: 10.1371/journal.pone.0134002 (PMC4532465; doi:10.1371/journal.pone.0134002)

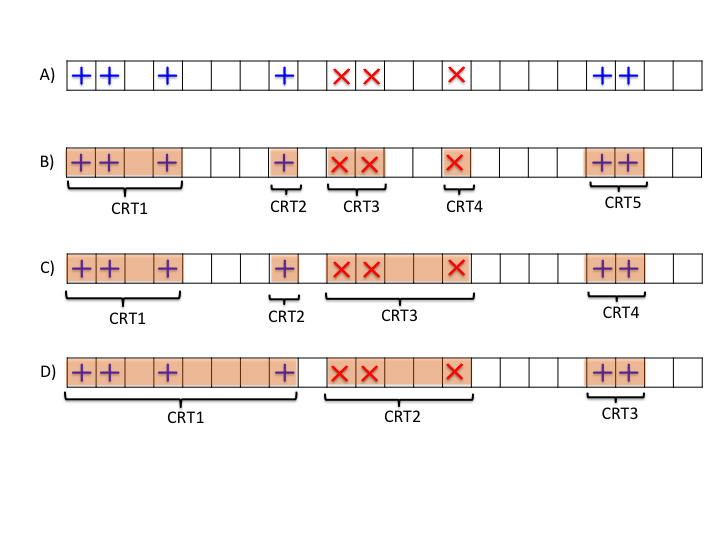

Supplement: S2 Fig — Each cell of the array corresponds to a time unit (tu). The two symbols (blue and red crosses) correspond to the detections at two different receivers. The data is processed with increasing MBP n following Eq (1) and taking ΔMBP = 1 tu. Raw data (A); Processed data with MBP 1 = 1 tu (B); MBP 2 = 2 tu (C) and MBP 2 = 3 tu (D). (PNG) [file pone.0134002.s005.png]

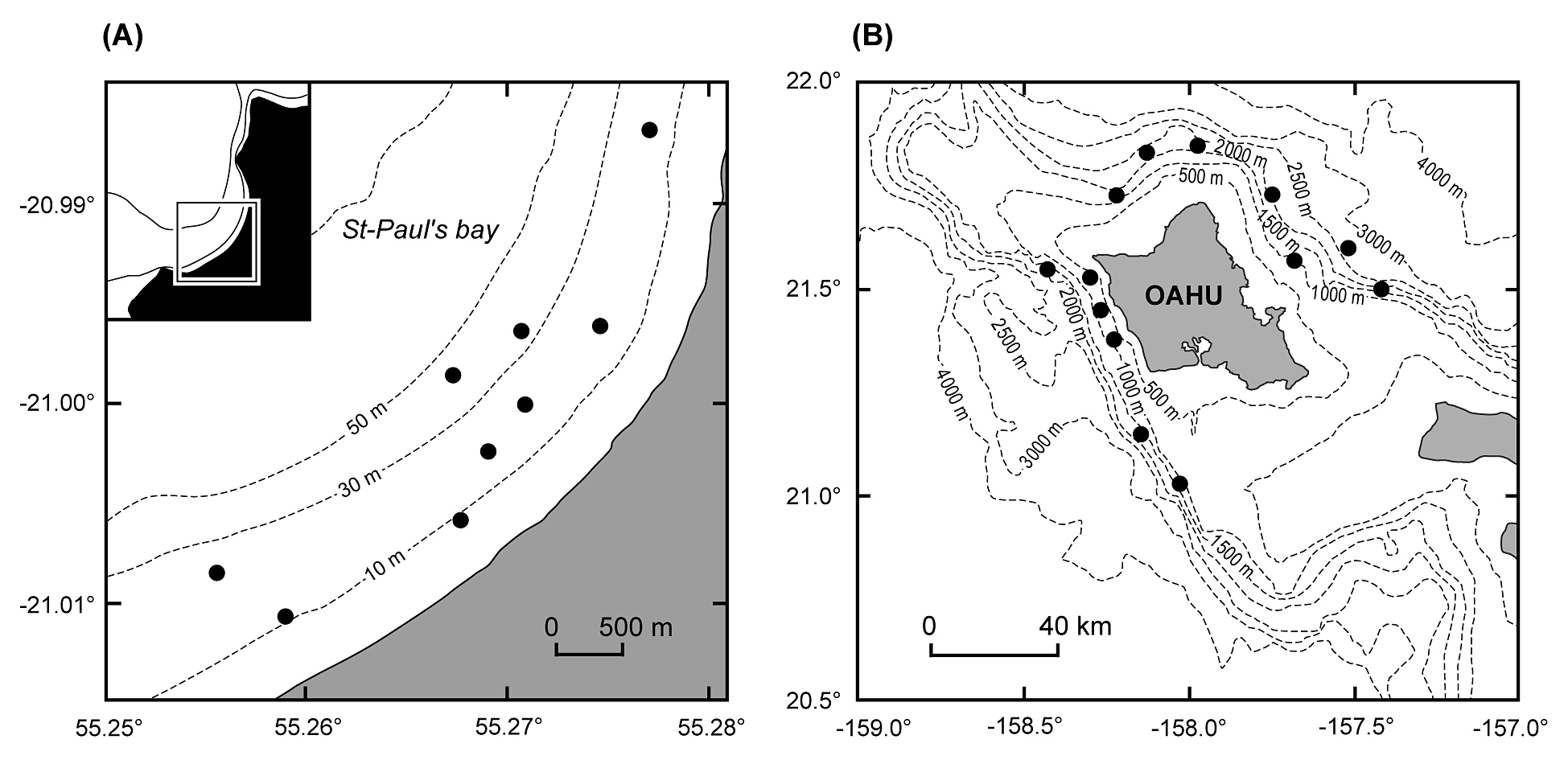

Supplement: S3 Fig — Each equipped FAD is represented by a black dot. (PNG) [file pone.0134002.s006.png]
